# Influence of Maternal Bifidobacteria on the Development of Gut Bifidobacteria in Infants

**DOI:** 10.3390/ph5060629

**Published:** 2012-06-18

**Authors:** Katsunaka Mikami, Moto Kimura, Hedenori Takahashi

**Affiliations:** 1 Deparment of Psychiatry, Tokai University School of Medicine, 143 Shimokasuya, Isehara, Kanagawa 259-1193, Japan; 2 Laboratory for Infectious Disease, Tokai University School of Medicine, 143 Shimokasuya, Isehara, Kanagawa 259-1193, Japan; 3 Sagami Research Laboratories, Wakamoto Pharmaceutical Co., LTD. 378 Kanate, Ohi-Machi, Ashigarakami-gun, Kanagawa 258-0018, Japan

**Keywords:** bifidobacteria, mother, infant, transmission

## Abstract

Intestinal microbiota plays an important role in human health by influencing metabolic activities that result in the creation of energy and absorbable nutrients, a barrier to the colonization of pathogens, and stimulation of the immune system. The development of fecal microbiota in neonates is crucial because those bacteria are the first to colonize the sterile intestine of the neonates and, thus, have a significant effect on the host. Initial colonization is also relevant to the final composition of the permanent microbiota in adults. Bifidobacteria are predominant in the fecal microbiota of infants, and, therefore, they are important to an understanding of how commensal bifidobacteria is established in the intestine of infants. While the mother’s bifidobacteria are considered to significantly influence the infant’s bifidobacteria, it is not clear whether a specific bifidobacterial strain transmits vertically from mother to infant and what factors of the mother before delivery influence the establishment of intestinal bifidobacteria in infants. This review focuses on the impact of maternal bifidobacteria on the development of gut bifidobacteria in the infant and suggests that there is cumulative evidence regarding bifidobacterial transfer from the maternal gut or breast milk to the infant gut.

## The Significance of Intestinal Bifidobacteria

Intestinal microbiota has a rich flora of more than 500 different bacterial species, some of which have important health-promoting functions. The number of microbial cells within the gut lumen is about 10 times larger than the number of eukaryotic cells in the human body [1]. Intestinal microbiota plays an important role in human health by: (1) initiating metabolic activities that result in the salvage of energy as short-chain fatty acids, the production of vitamin K, and the absorption of ions; (2) controlling epithelial cell proliferation and differentiation; (3) developing the immune systems; and (4) protecting against pathogens [2]. The development of fecal microbiota in neonates is critical because pioneer bacteria can modulate the expression of genes in host epithelial cells [3]. Initial colonization is also relevant to the final composition of the permanent microbiota in adults.

Bifidobacteria are predominant in the fecal microbiota of infants, and they are considered to be the most important physically and mentally beneficial bacteria for infants. Firstly, *Bifidobacterium breve* (*B. breve*) administered to preterm infants can up-regulate transforming growth factor beta 1, which activates mucosal regulation [4]. In addition, a randomized, double-blind, placebo-controlled trial found that administration of infant formula including *B. breve* for five months to healthy infants aged four to six months reduced the severity of acute diarrhea [5]. These studies found that gut bifidobacteria in infants play a crucial role in protecting against pathogens by keeping the immune and protective functions in epithelial cells. Second, probiotics, including bifidobacteria, might be related to allergies [6,7], and function in the prevention of allergic disease, particularly allergic dermatitis [8]. Thirdly, initial intestinal microbiota, and bifidobacterial colonization in particular, may be a prerequisite for the induction of oral tolerance on the basis of the investigation of the IgE response in germ-free mice [9]. Finally, commensal microbiota may affect the postnatal development of the hypothalamic-pituitary-adrenal (HPA) stress response in the early developmental stage in mice. The exaggerated HPA stress response of germ-free mice was reversed by the reconstitution of *Bifidobacterium infantis* (*B. infantis*) [10].

## Physiological Process of the Development for the Bifidobacterial Microbiota in Guts

The first bacteria colonizing the intestine in newborns delivered by vaginal delivery are of maternal origin and, primarily, facultative anaerobic bacteria colonized there immediately after birth. The environment in the intestine of neonates shows a positive redox potential at birth [11]. The gastrointestinal tract is, therefore, first colonized by facultative anaerobes, which lower the redox potential and then permit the growth of strict anaerobes, which normally appear at high levels during the first week of life [11]. Neonatal human intestines contain large numbers of facultative anaerobes or aerobes, such as *Staphylococcus*, *Enterobacteriaceae*, and *Streptococcus*. These facultative anaerobes or aerobes are transient and are soon replaced by obligate anaerobes, such as *Bifidobacterium*, *Bacteroides*, and *Clostridium*. Eventually, these obligate anaerobes become the predominant bacteria residing in the gut [12,13,14]. Approximately one month after birth, bifidobacteria are the most predominant fecal bacteria in both bottle-fed and breast-fed groups [15], and they remain in the intestine throughout infancy. In addition, bifidobacteria remain at a high rate in fecal bacteria until old age [16].

As for *Bifidobacterium* species in the infant gut, Matsuki *et al*. examined 27 breast-fed infants of approximately one month of age using the PCR method [17]. *B. breve* was the most frequently found species, followed by *B. infantis* and *Bifidobacterium longum* (*B. longum*). Mikami *et al*. [18] examined fecal samples of 110 mother-infant pairs using the PCR method. The feces collected from infants of approximately one month of age, 95% of whom were exclusively or partially breast-fed, were included in the analysis. *B. breve* was the most frequent species, followed by *B. longum* and *B. infantis*. In Finland, Grönlund *et al*. [19] examined 61 mother-infant pairs using the PCR method. Half of the mothers had received the supplementation of *Bifidobacterium lactis* and *Lactobacillus rhamnosus* (*L. rhamnosus*) from 15 weeks of gestation through delivery. In exclusively or partially breast-fed infants at the age of one month, the *B. longum* group (containing *B. longum* biotype *longum*, biotype *infantis,* and biotype *suis*) was the most frequent species, followed by *Bifidobacterium bifidum* (*B. bifidum*), *Bifidobacterium animalis* (*B. animalis*), and *B. breve* (the frequency of *B. bifidum* and *B. animalis* was equal). In Germany, Klessen *et al*. [20] examined 39 infants (human milk: 20, formula: 19) during three months of life using culture-dependent methods. In the group of infants aged one month and breast-fed, *B. infantis* was the most frequent, followed by *B. bifidum* and *B. breve*. This opposing result may be caused by differences in environmental and genetic backgrounds, but *B*. *breve*, *B. infantis*, and *B. bifidum* were the most popular species in infants at the age of one month worldwide.

## Influence of Non-Maternal Factors on the Establishment of Bifidobacteria in an Infant’s Guts

### Infant Feeding

In comparing the intestinal microbiota of breast-fed and formula-fed infants, there are high numbers of bifidobacteria in the former group, while the latter group is more diverse and contains more *Bacteroides*, *Clostridium*, and *Enterobacteriaceae* [13,15,20,21,22]. The mother’s breast milk contains a large amount of galacto-oligosaccharides as one of the main constituents, which selectively accelerate the growth of bifidobacteria [23,24,25]. Therefore, even if infants are fed using formulas, formulas including prebiotic oligosaccharides as a growth factor for bifidobacteria could lead to similar results as those seen in breast-fed infants. Haarman *et al*. [23] assessed the bifidobacterial ratio of total bacteria in infant guts using fully formula-fed term infants aged 28 to 90 days, suggesting that the percentage of bifidobacteria in breast-fed infants or infants fed formula supplemented with galacto- and fructo-oligosaccharides was significantly higher than that in the standard formula group after a six-week intervention period. 

### Horizontal and Vertical Transmission

After birth, the microbes from a mother’s mouth and skin and those of an environmental origin transfer horizontally to the newborn through several processes [13,26,27]. Moreover, frequent nosocomial intestinal acquisition of hospital strains of *E. coli* to neonates has been suggested using plasmid profiling [27].On the other hand, during birth through vaginal delivery, infants are first exposed to microbes that originate from the mother during the birthing process. Inoue *et al*. [28] analyzed fecal samples of 10 pups in the suckling stage and at maturity using an amplified ribosomal DNA restriction analysis to assess the mechanisms of bacterial transmission. Using amplified ribosomal DNA restriction analysis, the authors found that, although the diversity of intestinal microbiota during pup growth was influenced by horizontal transmission, vertically transmitted bacteria are the predominant component of intestinal microbiota. The proximity to the birth canal and the anus, as well as the parental expression of neonatal care, is an effective method of ensuring the transmission of microbes from one generation to the next [13]. For bifidobacteria that are one of the predominant bacteria in an adult’s gut [29], it is more important to primarily consider the influence of vertical transmission, in other words, the influence of maternal bifidobacteria.

## Influence of Maternal Bifidobacteria on the Establishment of Infant’s Bifidobacteria in the Gut

### Delivery Mode

Infants born by cesarean delivery can be exposed to their mothers’ microbiota, but initial exposure is most likely due to environmental isolates from equipment, air, other infants, and the nursing staff [13]. Grönlund *et al*. [30] examined the intestinal colonization of 64 healthy infants with different delivery mode until 180 days of age. Thirty-four infants were delivered vaginally, and 30, by cesarean section with antibiotic prophylaxis administered to their mothers before delivery. The frequency of the colonization of bifidobacterium-like bacteria was significantly smaller in the cesarean section delivery group until 10 days of age. Chen *et al*. [31] examined the intestinal colonization of 40 healthy breast-fed infants with different delivery methods for the first seven days after birth. Twenty infants were delivered vaginally, and 20, by cesarean section, with antibiotic prophylaxis administered to their mothers before delivery. The mean levels of bifidobacteria in the vaginally delivered group were significantly higher than those in the caesarean delivery group. Pender *et al*. [32] showed that cesarean delivery, compared with vaginal delivery, was associated with the decreased counts of gut bifidobacteria using a linear regression analysis. In our study [18], using a proportional odds logistic regression analysis, we determined that a cesarean section was significantly associated with decreases in both the counts and diversity of bifidobacteria in the infant gut. Given these results, it is well established that the type of delivery has a significant effect on the development of intestinal bifidobacteria in neonates.

### Influence of Maternal Bifidobacteria in Gut

Bifidobacteria are predominant in the fecal microbiota of infants and play an important role; therefore, it is important to understand how maternal intestinal or vaginal bifidobacteria influence the establishment of intestinal bifidobacteria in infants. As illustrated above, the gut bifidobacteria of infants born through a cesarean section is very different from that of the neonates from a vaginal delivery [18,30,31,32], indicating a significant role of the mother-to-infant transmission of intestinal or vaginal bifidobacteria in the development of neonatal intestinal microbiota. In vaginal delivery, infants are first exposed to microbes of the mother’s microbiota in the vagina or gut during the birthing process, and, therefore, the development of neonatal intestinal microbiota depends on the mother’s microbiota. However, it is not clear whether a specific bifidobacterial strain vertically transmits from mother to infant, and, if so, what factors of the mothers’ bifidobacteria before delivery influence the establishment of intestinal microbiota in their infants. Although there have been only a few studies, evidence of this phenomenon is accumulating.

Our study [18] demonstrated that the detection of *B. breve* in the mothers’ feces was significantly associated with increases in both the bifidobacterial counts and number of *Bifidobacterium* species in the babies’ feces. Grönlund *et al*. [33] assessed 80 mother-infant paired fecal samples to examine the influence of maternal intestinal bifidobacteria on the establishment of bifidobacteria colonizing the gut in infants using quantitative real-time PCR assays. Probiotics were given to each mother two months prior to delivery and during breast-feeding for two months. Fecal samples were taken from the mothers one month postpartum and from the infants at one and six months of age. This study found that the mothers’ colonization of *B. bifidum* or *B. breve* was significantly associated with increases in the number of *Bifidobacterium* species in the infants’ guts at both one and six months of age; however, the association between mothers’ colonization of *B. breve* and the higher number of *Bifidobacterium* species in the infants’ guts aged six months was marginally significant. According to these two studies, it is considered that the mothers’ colonization of *B. breve* could affect the infants’ bifidobacteria in their guts. In Finland, the influence of *B. bifidum* in maternal guts on the infants’ gut bifidobacteria was also suggested, and *B. bifidum* was one of the predominant *Bifidobacterium* species in infant [33] ; however, this was not the case in Japan [18]. This inconsistency in the results may be caused by differences in environmental and genetic backgrounds, which means that the presence of *B. breve* in Japan or *B. bifidum* in Finland plays an important role in gut bifidobacteria in the infant. Therefore, it is crucial to determine a key *Bifidobacterium* species in maternal guts in each country.

Our study [18] and Grönlund *et al*. [19] found that there was no significant correlation between the fecal bifidobacterial counts of the mothers and those of the infants. In a separate study, however, Grönlund *et al*. [33] showed that there was a significant correlation between the fecal bifidobacterial counts of the mothers and those of the infants. This study was a follow-up study involving only high-risk allergy mothers who have clinical symptoms of allergies, and, therefore, these subjects may lead to different results.

As we have found, maternal intestinal bifidobacteria influences the establishment of intestinal bifidobacteria in infants, and thus, it could be important to intervene with probiotics such as bifidobacteria or prebiotics such as oligosaccharide to increase pregnant women’s intestinal bifidobacteria. Shadid *et al*. [34] assessed 48 pregnant women supplemented three times daily with galacto- and fructo- oligosaccharides or a placebo formula from week 25 of gestation until delivery in a randomized, double-blind, placebo-controlled study. The percentages of bifidobacteria within total bacterial counts were detected by quantitative PCR in maternal and neonatal stool samples on day 5 and 20 and after six months. The study found that, in neonates, the bifidobacteria percentages, diversity, and similarity indexes did not differ significantly between the two groups. Gueimonde *et al*. [35] assessed 53 mother-infant pair samples to characterize both the mother-infant bifidobacteria transfer and the development of bifidobacteria microbiota at three and five weeks in infants. The study compared mothers receiving *L*. *rhamnosus* GG and those receiving a placebo regarding the transmission of intestinal bifidobacteria from mother to infant. Mothers started consumption of the placebo or *L*. *rhamnosus* four weeks before delivery and continued it until all fecal samples were collected. The study found that at five days of age, infants whose mothers received *L. rhamnosus* GG showed a significantly higher presence of *B. breve* and a lower one of *B. adolescentis* than those from the placebo group. In addition, *L. rhamnosus* GG consumption increased the bifidobacterial diversity in infants and reduced the bifidobacterial microbiota similarity between mother and infant. Similarly, Lahtinen *et al* [36] examined 122 mother-infant pair samples to assess the efficacy of prenatally administered probiotics from 36 weeks of gestation until delivery on intestinal colonization by particular *Bifidobacterium* species in infants. The study found that, at 90 days of age, the intestines of infants whose mothers received the probiotic during late pregnancy were more often colonized with the *B. longum* group (consisting of *B. longum* biotype *infantis*, *B. longum* biotype *longum*, and *B. bifidum*) than infants whose mothers received placebo, and there were trends toward increased prevalence of *B. breve* and decreased prevalence of *B. adolescentis* in the probiotic group. These results found that probiotic administration in women during late pregnancy benefits the early development of infant intestinal microbiota. These two studies indicate that probiotic *L*. *rhamnosus* GG administered to pregnant women results in an appropriate bifidobacterial mibrobiota in the maternal gut. Because there have been only a few studies on the intervention of probiotics such as bifidobacteria or prebiotics such as oligosaccharides in the intestine of pregnant women, it remains unclear whether the intervention of probiotics or prebiotics to the maternal intestine affects gut bifidobacteria in infants. 

### Transfer of Gut Bifidobacteria Strains from Mother to Infant

Although maternal bifidobacteria in guts may influence the intestinal bifidobacteria in the infant [18,33], the manner in which the species influneces the status of bifidobacteria in the infant remains unclear. One important question is whether intestinal *Bifidobacterium* strains vertically transmit from mother to infant. 

Table 1 shows the summary of studies on the transmission of *Bifidobacterium* strains from pregnant women’s gut or vagina or from mothers’ breast milk to their corresponding infant’s gut. Tannock *et al*. [37] examined isolates of *Enterobacteriaceae*, lactobacilli, and bifidobacteria cultured from vaginal, rectal, and oral swabs collected from women soon after admission to a maternity hospital and compared them with those of strains detected in the feces of their infants using a plasmid profiling method. Lactobacilli, bifidobacteria, or *Enterobacteriaceae* inhabiting the vagina of the mothers did not appear to colonize the infant’s digestive tract, but genetic evidence of the transmission of fecal isolates of *Enterobacteriaceae* and bifidobacteria from mother to infant was obtained in four of five cases.

Using the paired mother-infant samples, Takahashi *et al*. [38] examined isolates from 21 paired samples for *B. breve* and 34 paired samples for *B. longum* to evaluate the possibility of *B. breve* transfer during the perinatal period by the random amplification of polymorphic DNA (RAPD) [39]. The number of the isolates bearing a shared type with *B. breve* was about 10 times higher than that of those with *B. longum*, suggesting that *B. breve* may be frequently transferred from mothers to infants. Examination of the growth of *B. breve* strains revealed that shared type strains of mothers have a significantly higher growth than non-shared-type strains in the presence of galacto-oligosaccharides or at a higher redox potential. These results suggested that *B. breve* strains in the mothers may be transferred to their infants and that not all of the mothers’ *B. breve* strains were easy to transfer, although *B. breve* strains with a higher growth ability in the presence of galacto-oligosaccharides or more strongly resilient to redox potential could transfer more easily.

**Table 1:** Studies on the transmission of bifidobacterial strains from pregnant women to their infant’sgut.

Reference	No. of mother-infant paired samples	Age of infant	Detection of species	Methods	Route of transmission assessed	Results of transmission
Tanock *et al*. [37]	5	10 and 30 days	Not described in detail	Plasmid profiling	Vagina and gut	Found in gut but not in vagina
Solís *et al*. [55]	20	1, 10, 30, and 90 days	*B. breve*, *B. longum*	RAPD	Breast milk	Found but not described in detail
Takahashi *et al*. [38]	10 (*B. breve*), 8 (*B. longum*)	Between 3 and 6 weeks	*B. breve*, *B. longum*	RAPD	Gut	*B. breve* and *B. longum* found
Makino *et al*. [46]	8	0, 3, 7, 30, and 90 days	*B. longum* subsup. *longum*	MLST, AFLP	Gut and breast milk	Found in gut and breast milk

Abbreviations: RAPD, random amplification of polymorphic DNA; MLST, multilocus sequencing typing; AFLP, amplified fragment length polymorphism; N/A, not available.

It is important to sequence the entire genome in order to discriminate the vertical transmission of bifidobacteria from mother to infant at the strain level. RAPD [39], the amplified fragment length polymorphism (AFLP) [40], and ribotyping [41] have provided rapid identification of the strain level. Furthermore, recent DNA sequencers have achieved remarkable development and a lower cost of analysis. Multilocus sequence typing (MLST) could sequence a pattern of several genes in the genome and has the capacity for high resolution, which is suitable for both species identification and strain typing [42,43,44,45]. Makino *et al*. [46] assessed fecal samples from eight mothers before delivery and their breast-fed infants taken at 0, 3, 7, 30, and 90 days of age. This study investigated the mother-to-infant transmission of *B. longum* subsp. *longum* strains of 207 isolates from fecal samples (195 isolates) or breast milk (12 isolates) from mother-infant pairs using MLST and AFLP analysis. Eleven strains of *B. longum* subsp. *longum* were found to be monophyletic for the feces of the mother and her infant, confirming that these strains were transferred from the intestine of the mother to that of the infant. Furthermore, these strains were found in the first feces of the infant and in the feces at days 3, 7, 30, and 90 after birth, indicating that they were stable colonies in the infant’s intestine immediately after birth. The strains isolated from each family did not belong to clusters derived from any of the other families, suggesting that each mother-infant pair might have unique family-specific strains. These findings confirm that specific *Bifidobacterium* strains in guts transmit vertically from mother to infant, expand in numbers soon after birth, and subsequently colonize. There is accumulating evidence of the importance of mother-to-infant transmission of bifidobacteria and the colonization of infant gastrointestinal tracts. 

### Maternal Bifidobacteria in Vagina

The presence of bifidobacteria in the vagina has been reported using a culturally dependent or independent method [18,37,47,48,49]. Verhelst *et al*. [49] examined 515 vaginal swabs of 197 pregnant women. The authors described a specific vaginal microbiota category termed “grade I-like,” characterized by the Gram staining method, and established that half of these samples contained *Bifidobacterium* spp, primarily *B. breve*. Mikami *et al*. [18] examined the bifidobacterial species of 100 vaginal swabs of pregnant women. In this study, using PCR methods, bifidobacteria were detected in more than 80% of the vaginal swab samples, and *B. breve* was the most prevalent species, followed by the *B. catenulatum* group and *B. adolescentis*, while the count of total bifidobacteria was smaller than that of total *Lactobacilli*, as previously reported. Swidsinski *et al*. [50] investigated the putative concordance of the presence of *Gardnerella vaginalis* and a series of bifidobacteria between the perianal and vaginal microbiota in 10 patients with bacterial vaginosis through multicolor fluorescence *in situ* hybridization analysis of desquamated epithelial cells. In most women, at least one of the following species was detected perianally: *B. adolescentis*, *B. longum*, *B. breve*, *B. bifidum, or* *B. catenulatum*. At the vaginal site, none of these bifidobacteria was found.

The gut bifidobacteria of infants born by cesarean section are very different from those of the neonates from a vaginal delivery [18,30,31,32], indicating the significant role of the mother-to-infant transmission of vaginal bifidobacteria in the development of neonatal intestinal microbiota. However, to our knowledge, there have been few studies showing the transmission of the bifidobacterial strain from maternal vagina to guts in infants. Tannock *et al*. (36) examined isolates of bifidobacteria cultured from vaginal swabs collected from women soon after admission to a maternity hospital and compared them with those of strains detected in the feces of their infants using the plasmid profiling method. Bifidobacteria inhabiting the vagina of the mothers did not appear to colonize the infants’ digestive tract. In our study [18], there was no significant correlation between the bifidobacterial counts of the vaginal swabs and those of the babies’ feces. In addition, maternal bifidobacteria in the vagina were not associated with the bifidobacterial counts or the number of *Bifidobacterium* species in the infants’ feces using a proportional odds logistic regression analysis. 

### Maternal Bifidobacteria in Breast Milk

Recent studies have indicated that breast milk can be another source of intestinal microbes and that the presence of bifidobacterial DNA is a common event in breast milk [19,46,51,52,53,54,55]. Gueimonde *et al*. [51] assessed breast milk samples from 20 women with their one-month old infants to examine whether human milk contained bifidobacteria using a quantitative real-time PCR method. The *B. longum* group (*B. longum* biotype *longum*, *B. longum* biotype *infantis,* and *B. longum* biotype *suis*) was the most predominant species, followed by the *B. animalis*, *B. bifidum*, and *B. catenulatum* groups. Grönlund *et al*. showed the same rank of *bifidobacterium* species as in the study of Gueimonde *et al*., and the median count of the genus *Bifidobacterium* in breast milk was 1.4 x 10^3^ bacteria/ml. Martin *et al*. [52] assessed breast milk samples from 23 women with their infants between four and seven days after birth to isolate and identify bifidobacteria using a culture-dependent method or PCR-denaturing gradient gel electrophoresis and a quantitative real-time PCR method. *B. adolescentis* was the predominant species, followed by *B. longum*, *B. bifidum*, and *B. breve*. The mean count of the genus *Bifidobacterium* in breast milk was 3.35 x 10^3^ bacteria/mL.

It is necessary to determine whether bifidobacteria in breast milk transfer to the gut in the infant. In order to ascertain this event, the transfer of the bifidobacterial strains in breast milk to the infant gut has to be confirmed. Solís *et al*. [55] primarily assessed the establishment of lactic acid bacteria and bifidobacteria during the first three months of age in 20 vaginally delivered, breast-fed, full-term infants. In addition, the presence of viable *Bifidobacterium* strains in the corresponding breast-milk samples was evaluated using the RAPD method. The study found that bifidobacterial strains showing the same genetic profiles were present in breast milk and the corresponding infant feces at different sampling points (detail data not shown in the paper), suggesting vertical transfer from the mother's milk to the infant. Identical profiles were not found among isolates from different infants, indicating that during the first months of age, the numerically predominant bifidobacterial populations are individual-specific. Makino *et al*. [46] assessed the mother-to-infant transmission of *B. longum* subsp. *longum* strains in breast milk (12 isolates) taken at 7 and 30 days after delivery and fecal samples of the corresponding infants taken at 0, 3, 7, 30, and 90 days of age using MLST and AFLP analysis. The study suggested that two monophyletic strains of *B. longum* subsp. *longum* were transferred from the breast milk to the infant gut, confirming specific *Bifidobacterium* strains in breast milk transfer to the corresponding infant gut. Although several authors have reported the presence of bifidobacterial DNA in breast milk samples, its isolation seems to be more difficult because its concentration of breast-milk fluid is much lower, that is, about 10^3^ CFU/mL [19,52]. Martin *et al*. [52] were the first to succeed in isolating bifidobacteria. However, in only eight of 22 breast milk samples was DNA detected, suggesting a fastidious growth requirement of the bacteria present in this type of sample. Further investigations are under way to clarify the importance of bacterial transfer through breastfeeding. 

Although the vertical transmission of bifidobacteria in breast milk is evident, it has not been clear whether the bifidobacteria in breast milk influence the status of gut bifidobacteria in the infant. Grönlund *et al*. [19] found there has been no correlation between the total count of bifidobacteria in breast milk and that in the infants’ feces nor was there any association between the breast milk and infants’ fecal bifidobacteria colonization frequencies at the species level.

## Conclusions

There is cumulative evidence concerning bifidobacterial transfer from maternal gut or breast milk to the infant gut. It has been suggested that specific strains in the pregnant mother’s intestine transfer to the infant’s intestine, expand in numbers soon after birth, and, subsequently, colonize in the intestine of the infant. The detection of specific species, such as *B. breve* or *B. bifidum,* in maternal guts results in better bifidobacterial ecology in the corresponding infant’s gut. This depends on the differences in the environmental bacterial load and on the genetic background of the study population, and, therefore, it is important to identify appropriate bifidobacterial species better suited for each country. In addition, not all of the strains of *B. breve* or *B. bifidum* in maternal guts contribute to the bifidobacterial microbiota in the infant. For example, specific *Bifidobacterium* strains.with a higher growth ability in the presence of sensitivity to galacto-oligosaccharides or those more strongly resilient to the redox potential are easier to transfer to the infant gut. Further studies to detect more appropriate strains of bifidobacteria are needed in order to determine whether the intervention of those species in pregnant mothers should be considered as a probiotic. Factors that contribute to the ecology of bifidobacterial microbiota in guts in early life will continue to be explored because bifidobacteria are considered to be the most important physically and mentally beneficial bacteria for infants.

## References

[1] Bengmark S. (1998). Ecological control of the gastrointestinal tract. The role of probiotic flora. Gut.

[2] Guarner F., Malagelada J.R. (2003). Gut flora in health and disease. Lancet.

[3] Hooper L.V., Wong M.H., Thelin A., Hansson L., Falk P.G., Gordon J.I. (2001). Molecular analysis of commensal host-microbial relationships in the intestine. Science.

[4] Fujii T., Ohtsuka Y., Lee T., Kudo T., Shoji H., Sato H., Nagata S., Shimizu T., Yamashiro Y. (2006). Bifidobacterium breve enhances transforming growth factor beta1 signaling by regulating smad7 expression in preterm infants. J. Pediatr. Gastroenterol. Nutr..

[5] Thibault H., Aubert-Jacquin C., Goulet O. (2004). Effects of long-term consumption of a fermented infant formula (with bifidobacterium breve c50 and streptococcus thermophilus 065) on acute diarrhea in healthy infants. J. Pediatr. Gastroenterol. Nutr..

[6] Bjorksten B., Sepp E., Julge K., Voor T., Mikelsaar M. (2001). Allergy development and the intestinal microflora during the first year of life. J. Allergy Clin. Immunol..

[7] He F., Ouwehand A.C., Isolauri E., Hashimoto H., Benno Y., Salminen S. (2001). Comparison of mucosal adhesion and species identification of bifidobacteria isolated from healthy and allergic infants. FEMS Immunol. Med. Microbiol..

[8] Kukkonen K., Savilahti E., Haahtela T., Juntunen-Backman K., Korpela R., Poussa T., Tuure T., Kuitunen M. (2007). Probiotics and prebiotic galacto-oligosaccharides in the prevention of allergic diseases: A randomized, double-blind, placebo-controlled trial. J. Allergy Clin. Immunol..

[9] Sudo N., Sawamura S., Tanaka K., Aiba Y., Kubo C., Koga Y. (1997). The requirement of intestinal bacterial flora for the development of an ige production system fully susceptible to oral tolerance induction. J. Immunol..

[10] Sudo N., Chida Y., Aiba Y., Sonoda J., Oyama N., Yu X.N., Kubo C., Koga Y. (2004). Postnatal microbial colonization programs the hypothalamic-pituitary-adrenal system for stress response in mice. J. Physiol..

[11] Bezirtzoglou E. (1997). The intestinal microflora during the first weeks of life. Anaerobe.

[12] Caicedo R.A., Schanler R.J., Li N., Neu J. (2005). The developing intestinal ecosystem: Implications for the neonate. Pediatr. Res..

[13] Mackie R.I., Sghir A., Gaskins H.R. (1999). Developmental microbial ecology of the neonatal gastrointestinal tract. Am. J. Clin. Nutr..

[14] Rotimi V.O., Duerden B.I. (1981). The development of the bacterial flora in normal neonates. J. Med. Microbiol..

[15] Benno Y., Sawada K., Mitsuoka T. (1984). The intestinal microflora of infants: Composition of fecal flora in breast-fed and bottle-fed infants. Microbiol. Immunol..

[16] Mitsuoka T. (1992). Intestinal flora and aging. Nutr. Rev..

[17] Matsuki T., Watanabe K., Tanaka R., Fukuda M., Oyaizu H. (1999). Distribution of bifidobacterial species in human intestinal microflora examined with 16s rrna-gene-targeted species-specific primers. Appl. Environ. Microbiol..

[18] Mikami K., Takahashi H., Kimura M., Isozaki M., Izuchi K., Shibata R., Sudo N., Matsumoto H., Koga Y. (2009). Influence of maternal bifidobacteria on the establishment of bifidobacteria colonizing the gut in infants. Pediatr. Res..

[19] Gronlund M.M., Gueimonde M., Laitinen K., Kociubinski G., Gronroos T., Salminen S., Isolauri E. (2007). Maternal breast-milk and intestinal bifidobacteria guide the compositional development of the bifidobacterium microbiota in infants at risk of allergic disease. Clin. Exp. Allergy.

[20] Kleessen B., Bunke H., Tovar K., Noack J., Sawatzki G. (1995). Influence of two infant formulas and human milk on the development of the faecal flora in newborn infants. Acta Paediatr..

[21] Mevissen-Verhage E.A., Marcelis J.H., de Vos M.N., Harmsen-van Amerongen W.C., Verhoef J. (1987). Bifidobacterium, bacteroides, and clostridium spp. In fecal samples from breast-fed and bottle-fed infants with and without iron supplement. J. Clin. Microbiol..

[22] Harmsen H.J., Wildeboer-Veloo A.C., Raangs G.C., Wagendorp A.A., Klijn N., Bindels J.G., Welling G.W. (2000). Analysis of intestinal flora development in breast-fed and formula-fed infants by using molecular identification and detection methods. J. Pediatr. Gastroenterol. Nutr..

[23] Haarman M., Knol J. (2005). Quantitative real-time pcr assays to identify and quantify fecal bifidobacterium species in infants receiving a prebiotic infant formula. Appl. Environ. Microbiol..

[24] Knol J., Scholtens P., Kafka C., Steenbakkers J., Gro S., Helm K., Klarczyk M., Schopfer H., Bockler H.M., Wells J. (2005). Colon microflora in infants fed formula with galacto- and fructo-oligosaccharides: More like breast-fed infants. J. Pediatr. Gastroenterol. Nutr..

[25] Kunz C., Rudloff S., Baier W., Klein N., Strobel S. (2000). Oligosaccharides in human milk: Structural, functional, and metabolic aspects. Annu. Rev. Nutr..

[26] Fryklund B., Tullus K., Berglund B., Burman L.G. (1992). Importance of the environment and the faecal flora of infants, nursing staff and parents as sources of gram-negative bacteria colonizing newborns in three neonatal wards. Infection.

[27] Murono K., Fujita K., Yoshikawa M., Saijo M., Inyaku F., Kakehashi H., Tsukamoto T. (1993). Acquisition of nonmaternal enterobacteriaceae by infants delivered in hospitals. J. Pediatr..

[28] Inoue R., Ushida K. (2003). Vertical and horizontal transmission of intestinal commensal bacteria in the rat model. FEMS Microbiol. Ecol..

[29] Matsuki T., Watanabe K., Fujimoto J., Takada T., Tanaka R. (2004). Use of 16s rrna gene-targeted group-specific primers for real-time pcr analysis of predominant bacteria in human feces. Appl. Environ. Microbiol..

[30] Gronlund M.M., Lehtonen O.P., Eerola E., Kero P. (1999). Fecal microflora in healthy infants born by different methods of delivery: Permanent changes in intestinal flora after cesarean delivery. J. Pediatr. Gastroenterol. Nutr..

[31] Chen J., Cai W., Feng Y. (2007). Development of intestinal bifidobacteria and lactobacilli in breast-fed neonates. Clin. Nutr..

[32] Penders J., Thijs C., Vink C., Stelma F.F., Snijders B., Kummeling I., van den Brandt P.A., Stobberingh E.E. (2006). Factors influencing the composition of the intestinal microbiota in early infancy. Pediatrics.

[33] Gronlund M.M., Grzeskowiak L., Isolauri E., Salminen S. (2011). Influence of mother's intestinal microbiota on gut colonization in the infant. Gut Microbes.

[34] Shadid R., Haarman M., Knol J., Theis W., Beermann C., Rjosk-Dendorfer D., Schendel D.J., Koletzko B.V., Krauss-Etschmann S. (2007). Effects of galactooligosaccharide and long-chain fructooligosaccharide supplementation during pregnancy on maternal and neonatal microbiota and immunity--a randomized, double-blind, placebo-controlled study. Am. J. Clin. Nutr..

[35] Gueimonde M., Sakata S., Kalliomaki M., Isolauri E., Benno Y., Salminen S. (2006). Effect of maternal consumption of lactobacillus gg on transfer and establishment of fecal bifidobacterial microbiota in neonates. J. Pediatr. Gastroenterol. Nutr..

[36] Lahtinen S.J., Boyle R.J., Kivivuori S., Oppedisano F., Smith K.R., Robins-Browne R., Salminen S.J., Tang M.L. (2009). Prenatal probiotic administration can influence bifidobacterium microbiota development in infants at high risk of allergy. J. Allergy Clin. Immunol..

[37] Tannock G.W., Fuller R., Smith S.L., Hall M.A. (1990). Plasmid profiling of members of the family enterobacteriaceae, lactobacilli, and bifidobacteria to study the transmission of bacteria from mother to infant. J. Clin. Microbiol..

[38] Takahashi H., Mikami K., Nishino R., Matsuoka T., Kimura M., Koga Y. (2010). Comparative analysis of the properties of bifidobacterial isolates from fecal samples of mother-infant pairs. J. Pediatr. Gastroenterol. Nutr..

[39] Bingen E., Barc M.C., Brahimi N., Vilmer E., Beaufils F. (1995). Randomly amplified polymorphic DNA analysis provides rapid differentiation of methicillin-resistant coagulase-negative staphylococcus bacteremia isolates in pediatric hospital. J. Clin. Microbiol..

[40] Keim P., Kalif A., Schupp J., Hill K., Travis S.E., Richmond K., Adair D.M., Hugh-Jones M., Kuske C.R., Jackson P. (1997). Molecular evolution and diversity in bacillus anthracis as detected by amplified fragment length polymorphism markers. J. Bacteriol..

[41] Faruque S.M., Roy S.K., Alim A.R., Siddique A.K., Albert M.J. (1995). Molecular epidemiology of toxigenic vibrio cholerae in bangladesh studied by numerical analysis of rrna gene restriction patterns. J. Clin. Microbiol..

[42] Curran B., Jonas D., Grundmann H., Pitt T., Dowson C.G. (2004). Development of a multilocus sequence typing scheme for the opportunistic pathogen pseudomonas aeruginosa. J. Clin. Microbiol..

[43] Kotetishvili M., Stine O.C., Kreger A., Morris J.G. Jr., Sulakvelidze A. (2002). Multilocus sequence typing for characterization of clinical and environmental salmonella strains. J. Clin. Microbiol..

[44] Miller W.G., On S.L., Wang G., Fontanoz S., Lastovica A.J., Mandrell R.E. (2005). Extended multilocus sequence typing system for campylobacter coli, c. Lari, c. Upsaliensis, and c. Helveticus. J. Clin. Microbiol..

[45] Maiden M.C., Bygraves J.A., Feil E., Morelli G., Russell J.E., Urwin R., Zhang Q., Zhou J., Zurth K., Caugant D.A. (1998). Multilocus sequence typing: A portable approach to the identification of clones within populations of pathogenic microorganisms. Proc. Natl. Acad. Sci. USA.

[46] Makino H., Kushiro A., Ishikawa E., Muylaert D., Kubota H., Sakai T., Oishi K., Martin R., Ben Amor K., Oozeer R. (2011). Transmission of intestinal bifidobacterium longum subsp. Longum strains from mother to infant, determined by multilocus sequencing typing and amplified fragment length polymorphism. Appl. Environ. Microbiol..

[47] Levison M.E., Corman L.C., Carrington E.R., Kaye D. (1977). Quantitative microflora of the vagina. Am. J. Obstet. Gynecol..

[48] Redondo-Lopez V., Cook R.L., Sobel J.D. (1990). Emerging role of lactobacilli in the control and maintenance of the vaginal bacterial microflora. Rev. Infect. Dis..

[49] Verhelst R., Verstraelen H., Claeys G., Verschraegen G., Van Simaey L., De Ganck C., De Backer E., Temmerman M., Vaneechoutte M. (2005). Comparison between gram stain and culture for the characterization of vaginal microflora: Definition of a distinct grade that resembles grade i microflora and revised categorization of grade i microflora. BMC Microbiol..

[50] Swidsinski A., Dorffel Y., Loening-Baucke V., Mendling W., Schilling J., Patterson J.L., Verstraelen H. (2010). Dissimilarity in the occurrence of bifidobacteriaceae in vaginal and perianal microbiota in women with bacterial vaginosis. Anaerobe.

[51] Gueimonde M., Laitinen K., Salminen S., Isolauri E. (2007). Breast milk: A source of bifidobacteria for infant gut development and maturation?. Neonatology.

[52] Martin R., Jimenez E., Heilig H., Fernandez L., Marin M.L., Zoetendal E.G., Rodriguez J.M. (2009). Isolation of bifidobacteria from breast milk and assessment of the bifidobacterial population by pcr-denaturing gradient gel electrophoresis and quantitative real-time pcr. Appl. Environ. Microbiol..

[53] Martin R., Langa S., Reviriego C., Jiminez E., Marin M.L., Xaus J., Fernandez L., Rodriguez J.M. (2003). Human milk is a source of lactic acid bacteria for the infant gut. J. Pediatr..

[54] Perez P.F., Dore J., Leclerc M., Levenez F., Benyacoub J., Serrant P., Segura-Roggero I., Schiffrin E.J., Donnet-Hughes A. (2007). Bacterial imprinting of the neonatal immune system: Lessons from maternal cells?. Pediatrics.

[55] Solis G., de Los Reyes-Gavilan C.G., Fernandez N., Margolles A., Gueimonde M. (2010). Establishment and development of lactic acid bacteria and bifidobacteria microbiota in breast-milk and the infant gut. Anaerobe.

